# High *Clostridium difficile* Infection among HIV-Infected Children with Diarrhea in a Tertiary Hospital in Mwanza, Tanzania

**DOI:** 10.1155/2020/3264923

**Published:** 2020-08-24

**Authors:** Mwanaisha Seugendo, Aldofine Hokororo, Rogatus Kabyemera, Delfina R. Msanga, Mariam M. Mirambo, Vitus Silago, Uwe Groß, Stephen E. Mshana

**Affiliations:** ^1^Department of Pediatrics and Child Health, Weill Bugando School of Medicine, Mwanza, Tanzania; ^2^Department of Microbiology and Immunology, Weill Bugando School of Medicine, Mwanza, Tanzania; ^3^Institute of Medical Microbiology, University Medical Center Göttingen, Göttingen, Germany

## Abstract

*Clostridium difficile* causes a million of illnesses each year worldwide and can affect people of all ages. Limited data exist on the prevalence of *C*. *difficile* infections (CDI) among children below five years of age in developing countries. This study is aimed at determining the prevalence, associated factors, and outcome of the *Clostridium difficile* infection among children with diarrhea attending a tertiary hospital in Mwanza, Tanzania. Stool samples were collected and cultured anaerobically to isolate *Clostridium difficile*, followed by *C*. *difficile* toxin A and B assay and ribotyping. A total of 301 children with diarrhea were enrolled. A total of 22 (7.31%, 95% CI: 0.89-0.95) nonrepetitive stool samples were positive for *Clostridium difficile*. Eighteen (81%) of *C*. *difficile* isolates were toxigenic, and 16 (72.7%) had unknown ribotypes. Independent predictors of positive *C*. *difficile* were as follows: positive HIV status, hospital stay of more than four days, high stool leukocyte count, and watery stool. *Clostridium difficile*-positive children had significantly higher median duration of the diarrhea than those without *C*. *difficile*. Clinicians should consider *C*. *difficile* as a possible cause of diarrhea in children living in developing countries and institute appropriate management to prevent associated morbidities and mortalities. Furthermore, there is a need of joint effort to improve *C*. *difficile* diagnosis and surveillance in developing countries to establish the unknown epidemiology of CDI in these countries.

## 1. Introduction

*C*. *difficile* is a gram-positive, cytotoxin-producing anaerobic bacterium commonly existing in the large intestines of humans and animals without causing disease [1, 2]. *Clostridium difficile* is one of the known causes of the antibiotic-associated diarrhea [3, 4]. Worldwide, *Clostridium difficile* causes a million of illnesses each year and can affect people of all ages [5]. The incidence of *Clostridium difficile* infection (CDI) has rapidly increased since 1990, with a marked increase in mortality since 2000 [1].

There is little understanding of the epidemiology of CDI in children especially in the low- and middle-income countries (LMICs). Few studies exist in Africa regarding the magnitude of CDI; these studies have documented the prevalence to range from 4.9 to 8.6% [6–10]. Recently, the prevalence of CDI among adults and children with diarrhea in Mwanza has been documented to be 6.4% [9]. Colonization of *C*. *difficile* among healthy children has been reported in a number of surveys and has been found to range from 2 to 64%, with more colonization being reported in neonates and infants [11–13].

Use of antibiotics such as third-generation cephalosporins or ampicillin-sulbactam with an aminoglycoside has been documented to be strongly associated with CDI in children [14, 15]. Other factors that have been found to be associated with CDI in children include the following: inflammatory bowel disease (IBD) [16] and use of proton pump inhibitors [17]. Few studies have reported CDI among HIV-infected children; a study from Poland [18] observed only 3 cases (17%) of CDI among 18 HIV-infected children; in Tanzania, seven (77.8%) out 9 *C*. *difficile* cases were children; and HIV infection was an independent factor that was associated with the occurrence of CDI. No study has been done to investigate the role of HAART and CDI in children; however, a multicenter study which included about 1% of children documented that CDI incidence was higher among untreated HIV-seropositive individuals but the incidence significantly declined during HAART treatment [19].

There is limited information regarding CDI in developing countries especially in children. The control of *C*. *difficile* requires understanding the magnitude and factors associated with CDI. A previous study [9], which included both adults and children, involved 108 children; the sample size was small to provide clear epidemiology of CDI in children in the study setting. Therefore, this study has documented the magnitude and factors associated with CDI among 301 children from a tertiary hospital in Mwanza, Tanzania.

## 2. Material and Methods

### 2.1. Study Design, Study Area, Inclusion Criteria, and Sampling

This was a hospital-based analytical cross-sectional study with a follow-up component until diarrhea stopped. It was conducted at the Bugando Medical Centre (BMC), a tertiary hospital in Mwanza, Tanzania, between February and September 2016.

All children aged 2 months to 12 years admitted with diarrhea (regardless of duration of the diarrhea) during the study period were serially enrolled until the desired sample size was reached. All inpatients who developed diarrhea while in the ward were also eligible to be enrolled. Demographic and clinical variables were collected using a standardized data collection tool. Diarrhea was defined as per World Health Organization “Diarrheal disease. 2013” [20].

### 2.2. Laboratory Procedures

All laboratory investigations were done following recommended SOPs as per the Bugando Medical Centre accredited laboratory ISO15189 by Southern African Development Community Accreditation Service (SADCAS) accreditation body with registration number MD 002. Nonrepetitive stool samples from children were collected and taken to the laboratory within 2 hours of collection. The watery/mucoid part of stool samples was cultured on selective *Clostridium* agar (BioMérieux, Paris, France) and incubated in the anaerobic condition (Anaerobic Pack BioMérieux, Marcy l'Etoile, France) for 24 hours as previously described [21].

Presumptive colonies of *C*. *difficile* were tested for glutamate dehydrogenase and *C*. *difficile* toxins A and B using rapid commercial tests (Quik Chek Complete, Alere TechLab, Blacksburg, VA, USA). Furthermore, multiplex PCR was done to detect toxin gene profiles [22] and PCR ribotyped by agarose or capillary gel electrophoresis was done to establish ribotypes as previously described [23]. In addition, stool was examined for leukocytes as previously described [24].

HIV testing was done according to the Tanzania national algorithm [25] using the SD Bioline HIV rapid test (Standard Diagnostic Inc., Korea) as the first test followed by Uni-Gold (Trinity Biotech, Ireland) as the second test. For children below 18 months, PCR was used to confirm HIV status as per National AIDS Control Programme (NACP) recommendations [25].

All patients enrolled in the study were assessed and managed as per BMC treatment guidelines as well as per WHO protocols. The antibiotic treatment in the pediatrics department includes ampicillin and gentamicin as the first line and third-generation cephalosporin as the second line. All children who were *C*. *difficile* positive were treated by metronidazole as recommended by South Africa treatment guidelines for CDI [26].

Children were followed, and the duration taken for when diarrhea stopped was recorded.

### 2.3. Data Analysis

Data were entered in the computer using Excel 2007 and cleaned and analyzed using STATA version 13 (College Station, Texas). Data were summarized in frequency tables and bar charts. Continuous variables (age and duration of when diarrhea stopped) were summarized as medians, while categorical variables were summarized as proportions. In this study, we defined *C*. *difficile* infection (CDI) as the acute onset of diarrhea with documented toxigenic *C*. *difficile* or its toxin or the isolation of *C*. *difficile* from the child with clinical manifestation [27]. To determine factors associated with *C*. *difficile*, backward univariate followed by multivariate logistic regression analyses were performed. All factors with *P* value less than 0.05 on univariate analysis were subjected on the multivariate logistic regression analysis. Cox regression analyses with Kaplan-Meier curves were used to assess the hazard ratio in relation to the outcome (diarrhea stopped). The 95% confidence interval was determined, and predictors with *P* value of less than 0.05 were considered to be statistically significant.

### 2.4. Ethical Considerations

The protocol of this study was approved by the Joint CUHAS-Bugando Ethics and Scientific Review Committee. Written Informed consent for the participation in the study was obtained from the mother/guardians of the respective child.

## 3. Results

### 3.1. Sociodemographic and Clinical Features

The median age of participants was 12 [IQR: 6-24] months, and approximately 156 (51.8%) of the children were males, and 164 (54.5%) of the enrolled children were below 12 months. Among 301 children enrolled into this study, 266 (88.4%) had history of using antibiotics before enrollment. The most frequently used antibiotics were as follows: amoxicillin, ampicillin, trimethoprim/sulfamethoxazole, erythromycin, and metronidazole. A total of 33 (11%) children were HIV positive (Table 1).

### 3.2. Prevalence and Factors Associated with *C*. *difficile*

Out of 301 children with diarrhea, 22 (7.3%, 95% CI: 4.4-10.2) had positivite *C*. *difficile* in their stool, hence possible CDI. All infected children were below 5 years of age. Other pathogenic bacteria detected from the stool were *Salmonella* spp. (19, 5.7%) and *Shigella* spp. (6, 1.9%). No patient had coinfection with *C*. *difficile*.

Out of 151 participants who presented with watery stool, 21 (13.9%) were *C*. *difficile* positive compared to 0.7% of 150 participants with loose pasty stool (*P* = 0.002). HIV-positive children had significantly more often *C*. *difficile*-positive stool samples than HIV-negative children (24.2% vs. 5.2%, *P* < 0.001). We also identified *C*. *difficile* significantly more often in patients who had a prolonged median hospital stay before diarrhea started than in those with a shorter median hospital stay (4.5 [4-8] days vs. 3 [1-7] days, *P* = 0.026). *Clostridium difficile*-positive children had significantly higher median duration (days) of diarrhea than those without *C*. *difficile* (6 [4-10] vs. 3 [2-5], *P* = 0.001).

Factors that independently predicted CDI were as follows: being HIV positive (OR: 7.9, 95% CI: 1.86-33.5, *P* = 0.005), watery stool (OR: 21.4, 95% CI: 1.3-343.8, *P* = 0.003), median hospital stay before diarrhea (OR: 1.12, 95% CI: 1.01-1.23, *P* = 0.026), median stool leukocytes (OR: 1.05, 95% CI: 1.02-1.23, *P* < 0.001), and median duration of diarrhea (OR: 1.16, 95% CI: 1.05-1.3, *P* = 0.003) (Table 2).

### 3.3. *C*. *difficile* Ribotypes, Toxin A, and Toxin B in 13 Strains That Were Typed

The majority (9/13 (69%)) of *C*. *difficile* isolates had unknown ribotypes. Toxin A and toxin B were detected in 9/13 of *C*. *difficile* isolates typed. Ribotypes 38 and 84 were detected in one isolate each and ribotype 45 in 2 isolates (Table 3).

### 3.4. Outcome in relation to When Diarrhea Stopped

**Figure 1:**
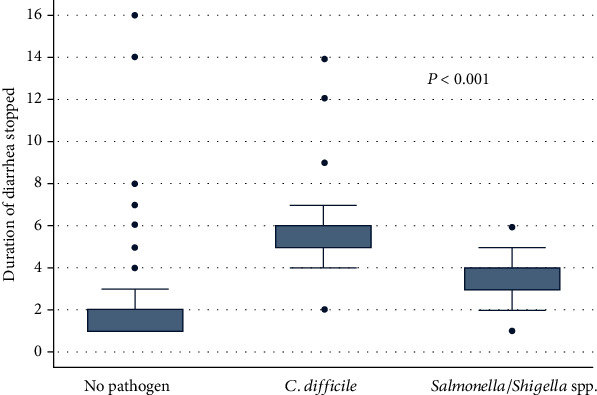
Median duration of when diarrhea stopped in 301 children (no pathogen, *C*. *difficile*, and *Salmonella* spp./*Shigella* spp.).

**Figure 2:**
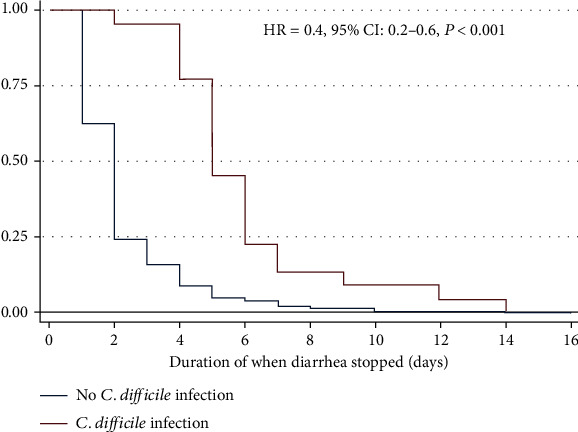
Kaplan-Meier curve showing the rate of when diarrhea stopped between CDI positive and CDI negative.

The outcome of CDI as measured by duration of when diarrhea stopped was determined. Children with CDI had significantly higher median duration of diarrhea than those without CDI (6 [4-10] days vs. 2 [2-4] days, *P* ≤ 0.001) (Figure 1). Based on Cox regression analysis, the rate of stopping diarrhea was significantly fast in children without CDI (HR: 0.4, 95% CI: 0.2-0.6, *P* < 0.001) (Figure 2).

## 4. Discussion and Conclusion

This study has observed the prevalence of CDI to be 7.3% among children with diarrhea which is comparable with previous studies in Tanzania (6.4%), Zimbabwe (8.6%), South Africa (7.1%), and Brazil (6.7%) [6, 7, 9, 28]. Comparing with other studies outside Africa (India (15%) and South Korea (13.2%)) [29, 30], the observed prevalence in the current study is significantly low which could be explained by fact that the studies with high prevalence used enzyme immunoassay (EIA) to detect cytotoxin; EIAs have been found to be more sensitive than standard culture technique and rapid toxin assays which were used in the current study [27]. Generally, in developed countries, among hospitalized children, the incidence of CDI follows an increasing trend necessitating more studies in developing countries to monitor the trend of CDI [31].

No coinfection between *C*. *difficile* and other organisms was observed in the current study which is different from other studies in South Africa, India, the United Kingdom, and Germany where coinfection with *Shigella* spp. and *Salmonella* spp. was linked to severe disease, high chances of complications, and reoccurrences [2, 7, 29, 32, 33].

In this study, circulating ribotypes included 038, 045, and 084 with majority of unknown ribotypes observed in our patients. The ribotype 084 has been recently found to be the predominant ribotype in Ghana [8, 10]. Most of the isolates typed in this study were toxigenic cementing their role in causing diarrhea in symptomatic children as previously documented [34].

In this study, we assessed the association between comorbidities and CDI [35]; we confirmed that positive HIV status was significantly associated with CDI similar to the findings from the previous study which involved both adults and children [9]. A study in South Africa did not find the association between CDI and HIV infection, and this was due to the fact that the HIV status was not known in a significant proportion of participants resulting in a low number of HIV-positive individuals in the subanalysis [7]. The findings regarding the association between HIV and CDI will help clinicians to have a high index of suspicion of CDI among HIV-infected individuals with history of hospitalization and antibiotic use. In addition, clinicians should also note that HIV-infected individuals are prone to opportunistic infections (OIs) requiring these patients to have frequent or prolonged courses of antimicrobial therapy contributing to the risk of CDI. Furthermore, studies have clearly shown the decrease in the incidence of CDI with HAART use [36].

Hospital stay for more than 5 days was significantly associated with CDI; similar observations were made by studies from India and Malaysia, which documented that patients who stayed in hospital settings for longer duration were more likely to acquire CDI [29, 37]. It was further observed that children with CDI significantly had longer hospital stay than those with no CDI [3, 35, 38]. CDI has been found to be associated with the local inflammatory process and systemic response [39], hence serious disease. This was confirmed in the current study whereby children with CDI had significantly more leukocytes in stool than those without CDI. *Clostridium*-positive children had significant longer duration of diarrhea than those without *C*. *difficile* and those infected with other organisms. This was also observed in a study in Poland [35] which showed a minimum of 3-4 days of recovery after starting treatment of CDI, but few cases had prolonged diarrhea of up to 6 days. In the USA [40], children with CDI had median hospital stay of 5.5 days similar to what has been observed in the present study. Another study in the USA observed increased pediatric CDI-related hospitalizations [41].

In the present study, in contrast to previous studies which showed a significant association between CDI and antibiotic use [9, 16, 27], a nonsignificant association was observed in the current study. This could be explained by the fact that the majority (88%) of children had history of using antibiotics providing unequal distribution of the outcome in different groups. We observed only one child to be CDI positive among 35 children with no history of antibiotic use.

One of the limitations of this study is the lack of a comparative group because asymptomatic infection has been observed in children [31, 42]; however, the study has provided information regarding CDI in these children with diarrhea and associated factors. It should be noted that to mitigate this limitation, the study excluded children below 2 months who are prone to be colonized with *C*. *difficile* [34, 43].

## 5. Conclusion

Toxigenic *Clostridium difficile* infections are prevalent among children below five years of age with diarrhea, and these infections are independently predicted by positive HIV status, prolonged hospital stay of more than four days, high leukocytes in stool, and watery stool. In addition, CDI was found to be associated with prolonged duration of diarrhea. Clinicians should consider CDI in children with diarrhea and initiate appropriate management to prevent associated morbidities and mortalities. *C*. *difficile* diagnosis and surveillance is urgently needed in developing countries to establish the unknown epidemiology of CDI in these countries.

## Figures and Tables

**Table 1:** Sociodemographic and clinical characteristics of 301 children with diarrhea.

Study variable	Number of patients	Percent
Age in months		
≤12 months	164	54.5
>12 months	137	45.5
Sex		
Male	156	51.8
Female	145	48.2
Type of stool		
Watery	151	50.2
Loose pasty	150	49.8
Residence		
Urban	194	64.5
Rural	107	35.5
HIV status		
Positive	33	10.96
Negative	268	89.04
Antibiotic use		
Yes	266	88.4
No	35	11.6
Type of toilet use		
Flush	115	38.2
Pit	186	61.8
Type of water source		
Tap	205	68.1
Nontap	96	31.9
Dehydration status		
No	158	52.5
Some	111	36.9
Severe	32	10.6
Parental income		
0-500K	223	74.09
500K-1M	60	19.93
>1M	18	5.98
Herbal use		
Yes	176	58.5
No	125	41.5

**Table 2:** Factors associated with CDI among study population (*n* = 301).

Variable	*C*. *difficile* infection		Univariate		Multivariate	
	Yes (%)	No (%)	OR [95% CI]	*P* value	OR [95% CI]	*P* value
Sex						
Male	12 (7.7)	144 (92.3)	1.0			
Female	10 (6.9)	135 (93.1)	0.9 [0.4-2.1]	0.791		
Age in months						
≤12months	9 (5.5)	155 (94.5)	1.0			
>12months	13 (9.5)	124 (90.5)	1.8 [0.7-4.3]	0.189		
Residence						
Urban	13 (6.7)	181 (93.3)	1.0			
Rural	9 (8.4)	98 (91.6)	1.3 [0.5-3.1]	0.586		
Antibiotic use						
No	1 (2.9)	34 (97.1)	1.0			
Yes	21 (7.9)	245 (92.1)	2.9 [0.4-22.4]	0.304		
HIV status						
Negative	14 (5.2)	254 (94.8)	1.0			
Positive	8 (24.2)	25 (75.8)	5.8 [2.2-15.2]	<0.001	7.9 [1.86-33.5]	0.005
Median diarrhea frequency (days)	9 [8-10]	4 [3-7]	1.4 [1.2-1.5]	<0.001	1.04 [0.82-1.32]	0.753
Type of stool						
Loose pasty	1 (0.7)	149 (99.3)	1.0	0.002	21.4 [1.3-343.9]	0.003
Watery	21 (13.9)	130 (86.1)	24.1 [3.2-181.1]			
Comorbidities						
No	7 (7.7)	84 (92.3)	1.0			
Yes	15 (7.1)	195 (92.9)	0.9 [0.4-2.3]	0.866		
Median hospital stay before diarrhea (days)	4.5 [4-8]	3 [1-7]	1.1 [1.01-1.14]	0.024	1.12 [1.01-1.23]	0.026
Median body temperature	38.2 [37.9-39]	37 [36.0-37.5]	2.8 [1.8-4.2]	<0.001	1.4 [0.9-2.5]	0.173
Median stool leukocytes	55 [45-70]	0 [0-4]	1.06 [1.04-1.08]	<0.001	1.05 [1.02-0.07]	<0.001
Median diarrhea duration	6 [4-10]	3 [2-5]	1.11 [1.04-1.18]	0.001	1.16 [1.05-1.30]	0.003

**Table 3:** Characteristic of *C*. *difficile* ribotypes (*n* = 13).

SNO	Strain	Age (months)	Ribotype	ToxA	ToxB
1	017	11	Unknown	NEG	NEG
2	O73	8	084	NEG	NEG
3	100	6	Unknown	POS	POS
4	171	60	Unknown	POS	POS
5	210	11	Unknown	NEG	NEG
6	221	24	Unknown	POS	POS
7	268	8	Unknown	POS	POS
8	270	21	Unknown	POS	POS
9	033	12	Unknown	POS	POS
10	091	15	Unknown	POS	POS
11	018	2	038	NEG	NEG
12	015	60	045	POS	POS
13	022	13	045	POS	POS

## Data Availability

The majority of data have been included in the manuscript; other data used to support the findings of this study are available from the corresponding author upon request.
